# Implantation, orientation and validation of a commercially produced heart-rate logger for use in a perciform teleost fish

**DOI:** 10.1093/conphys/coaa035

**Published:** 2020-04-23

**Authors:** Cuen Muller, Amber-Robyn Childs, Murray I Duncan, Michael R Skeeles, Nicola C James, Kerry-Ann van der Walt, Alexander C Winkler, Warren M Potts

**Affiliations:** 1 Department of Ichthyology and Fisheries Science, Rhodes University, Prince Alfred Street, PO Box 94, Makhanda 6140, South Africa; 2 South African Institute for Aquatic Biodiversity (SAIAB), Somerset Street, Makhanda 6140, South Africa

**Keywords:** Biologging, electrocardiogram, heart rate, *Pachymetopon grande*, sea bream, Sparidae

## Abstract

Quantifying how the heart rate of ectothermic organisms responds to environmental conditions (e.g. water temperature) is important information to quantify their sensitivity to environmental change. Heart rate studies have typically been conducted in lab environments where fish are confined. However, commercially available implantable heart rate biologgers provide the opportunity to study free-swimming fish. Our study aimed to determine the applicability of an implantable device, typically used on fusiform-shaped fish (e.g. salmonids), for a perciform fish where morphology and anatomy prevent ventral incisions normally used on fusiform-shaped fish. We found that ventrolateral incisions allowed placement near the heart, but efficacy of the loggers was sensitive to their orientation and the positioning of the electrodes. Electrocardiogram detection, signal strength and subsequent heart rate readings were strongly influenced by logger orientation with a significant effect on the quality and quantity of heart rate recordings. We provide details on the surgical procedures and orientation to guide future heart rate biologger studies on perciform-shaped fish.

## Introduction

Measurement of the heart rate (*f*_H_) of fish can provide estimates of metabolic rate and energy expenditure (Lucas *et al*. 1991; 1993; Cooke *et al*. 2010) and serve as an indicator of stress in response to different environmental conditions and human-induced disturbances (Schreer *et al*. 2001; Graham and Cooke 2008; Brijs *et al*. 2019). As such, quantifying how the *f*_H_ of fish responds to environmental stressors like temperature is important to accurately predict how ectothermic organisms will respond to environmental change. Increases in metabolic rates drive a rise in oxygen demand, which relates to an increase in cardiac output (Farrell *et al*. 2009; Ekström *et al*. 2018). This measure of increased cardiac activity, which may relate to food acquisition or predator avoidance, is a promising aspect for the refining of bioenergetics models (Cooke *et al*. 2016). Acquiring remote measurements from wild fish fitted with *f*_H_ loggers could provide important insights into how warming or upwelling events or variability in temperature in general affects foraging success, activity, behaviour and habitat use at an individual level (Cooke *et al*. 2012; Ekström *et al*. 2018). Autonomic regulation, for example, modulates *f*_H_ in response to temperature to meet oxygen demands, but at critical thermal maxima, heart rate is shown to plateau and subsequently decrease indicating that the delivery of oxygen is compromised at critical temperatures (Anttila *et al*. 2014; Skeeles *et al*. 2020). Individual differences in thermal tolerance as well as the mechanisms involved (e.g. coronary perfusion) may be important selective factors associated with warming (Ekström *et al*. 2019).

Weak correlations between *f*_H_ and metabolic rate in some instances have led to criticism in the use of *f*_H_ telemetry (Thorarensen *et al*. 1996; Butler *et al*. 2004). This weak relationship is primarily attributed to the varying contribution of stroke volume to total cardiac output, which may be a regulatory mechanism for a number of species (Clark *et al*. 2005). Nonetheless, as more species are found to predominantly regulate cardiac output through changes in *f*_H_ (Schreer *et al*. 2001; Cooke *et al*. 2002; Clark *et al*. 2005; Cooke *et al*. 2010) the method is well situated to provide important information on the physiology, ecology and energetics of wild fish (Cooke *et al*. 2004, 2016). Species-specific investigations into the relationships among cardiac activity and oxygen consumption rates should however be explored prior to field applications (Cooke *et al*. 2016).

Modern, fully implantable devices are leadless, lightweight and small; have sufficient battery power and data storage for months to years of observations; and are minimally invasive, with little to no hydrodynamic or behavioural modifications (Butler *et al*. 2004). These have been used for detecting the heart rate of a range of free-swimming fishes, particularly the salmonids *Oncorhynchus nerka* (Clark *et al*. 2009, 2010; Prystay *et al*. 2017), *O. kisutch* (Donaldson *et al*. 2010) and *O. mykiss* (Brijs *et al*. 2018; Ekström *et al*. 2018; Brijs *et al*. 2019) and gadoids *Gadus morhua* (Bjarnason *et al*. 2019; Davidsen *et al*. 2019). Up to now, there are few published studies on the use of these devices in perciform fishes (but see Prystay 2018; Prystay *et al*. 2019; Skeeles *et al*. 2020). One of the reasons for this may be the difficulty of implanting these loggers to effectively detect heart rate. This may partly be attributed to the morphology of these fishes, which have anteriorly positioned pectoral and pelvic girdles, making placement near the pericardium difficult, a narrow ventral surface, which reduces the opportunity for a ventral incision, and a deep body cavity, which encourages movement of the logger after placement. Since the orientation of the electrode on loggers has been shown to influence the accuracy of data collected (Brijs *et al*. 2018), the identification of optimal methods to place and maintain the loggers in position in the body cavity of perciforms is necessary. Using an archetypal perciform fish, the aim of this study was to describe an optimal procedure for the implantation and attachment of a commercially available *f*_H_ logger and to compare its efficacy when placed at different orientations in both anaesthetized and captive free swimming fish.

## Methods

### Study species, surgery and instrumentation

**Figure 1:**
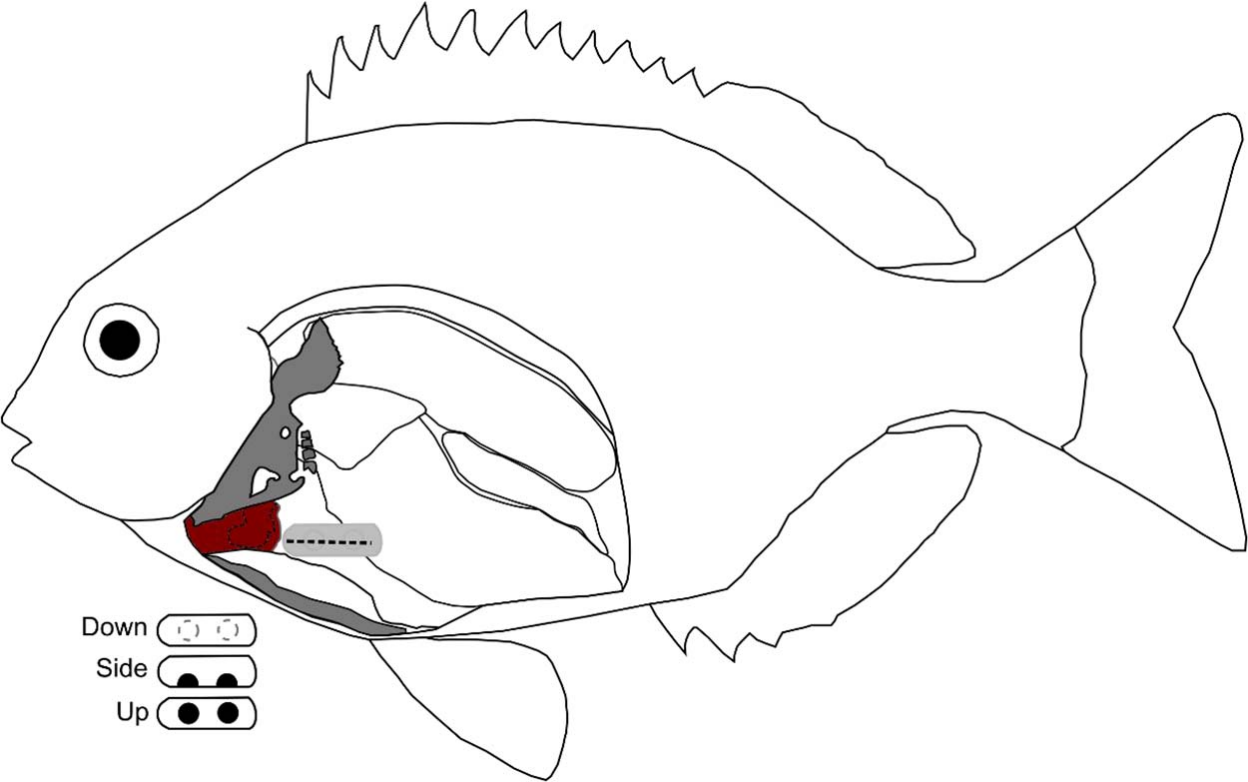
Proximity of biologger and incision (stippled line) relative to the pericardium (red), pelvic and pectoral girdles (grey) and within the abdominal cavity for *Pachymetopon grande*, a typical sparid. Three logger orientations tested: down—electrodes faced 180° away from incision; side—electrodes positioned ventrally at 45°; and up—electrodes positioned towards incision

Bronze bream (*Pachymetopon grande*; Günther, 1859; Sparidae) are endemic to southern Africa occurring in shallow, rocky coastal areas. Their oblong, slightly compressed body is typical of other sparids, an important Perciformes family of both commercial and recreational importance that is represented by approximately 29 genera and over 100 species worldwide (Heemstra and Heemstra 2004). For this study, *P. grande* were collected near Port Alfred (Eastern Cape, South Africa) with rod and line (permit number RES2019/76) and transported to the nearby NRF-SAIAB Aquatic Ecophysiology Research Platform (AERP) at Rhodes University, Makhanda (formerly Grahamstown). Prior to experiments, fish were held for a minimum of 1 month in 5900-L tanks operating on a saltwater recirculation system. Water temperature and light periodicity were controlled and reflected natural conditions at the time of capture; temperature was maintained at ~18°C and light:dark cycle 10:14. An anaesthetized baseline study was initially carried out on 10 restrained fish (length range 278–360 mm Fork Length), followed by an active, free-swimming treatment (*N* = 18, 260–368 mm FL). The aim of the baseline study was to determine whether logger position and orientation would be maintained with a double suture wrap (see Skeeles *et al*. 2020) while fish had limited mobility. Position and orientation of the logger were confirmed upon retrieval of the device at the completion of each trial. Following the baseline study, fish were used in a free-swimming treatment where logger orientation was randomly assigned at the time of surgery. Three logger orientations were investigated; down—electrodes faced 180° away from the ventrolateral incision and were in contact with the intestine; side—electrodes positioned at a 45° angle ventrally and in direct contact with musculature; and up—electrodes positioned towards the incision and were in contact with musculature (Fig. 1).

**Figure 2:**
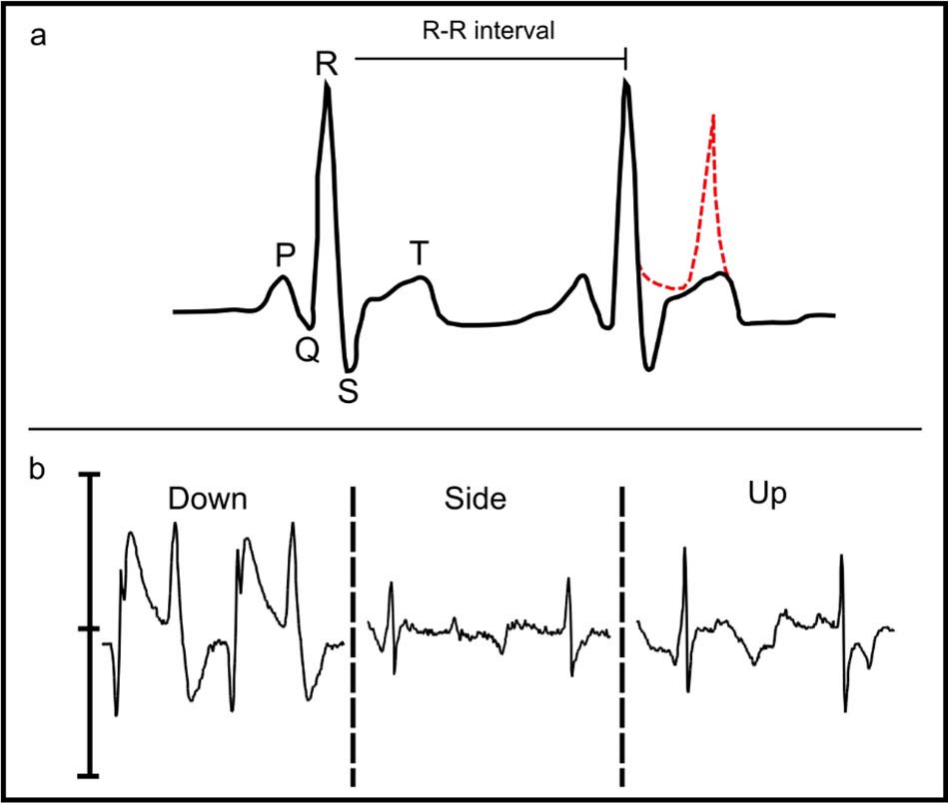
Conceptual electrocardiogram (ECG) at top (**a**) indicating elements associated with electrical conductivity where P wave indicates atrial contraction, QRS complex ventricular contraction and T wave ventricular relaxation. Poor electrode positioning may result in elevated T waves (stippled red line) which are falsely detected as R waves. Raw ECG waves from the three logger positions are shown below (**b**), note elevated T wave of down position

Biologgers used in the study were DST micro-HRT (L = 25.4 mm, W = 8.3 mm, Mass = 3.3 g, Star-Oddi, Iceland) heart rate and temperature loggers. The compact, lightweight devices monitor heart rate via a single-channel electrocardiogram (ECG) amplifier that uses two electrodes incorporated into the ceramic, leadless casing. Heart rate values are derived from mean R–R intervals, the positive peak in the QRS complex associated with ventricular depolarization (Fig. 2a), which is captured by burst measurements of ECG at set time intervals (Cooke *et al*. 2016; Bjarnason *et al*. 2019). Bursts are graded with a quality index (QI) which is rated from 0 to 3, ranging from great to poor (see: DST Micro-HRT User Manual). A temperature sensor is also located within the casing with an accuracy of 0.2°C as well as a real-time clock with an accuracy of 1 min. Captured measurements of *f*_H_ in BPM, as well as QI, are stored independently (ECG discarded) to reduce memory requirements and extend battery life. Loggers were programmed to record *f*_H_ at 15-s intervals at a frequency of 200 Hz, while ECG was recorded at 1-min intervals for a selected number of individuals for later validation of *f*_H_ readings, using the application software Mercury v 4.84 and the associated Communication Box (STAR-ODDI, Gardabaer, Iceland).

Fish were anaesthetized prior to surgery with 2-phenoxyethanol at a dose of 0.2 mL L^−1^ (Priborsky and Velisek 2018) until the loss of equilibrium, upon which they were placed in an operating sling lateral side up. A soft wet microfibre cloth covered the eyes while anaesthetic water was poured over the gills and cloth to maintain anaesthesia and keep external organs moist. Surgery involved a single ventrolateral incision immediately posterior of the pectoral girdle of ~25 mm. The logger was placed within the body cavity, posterior to but in contact with the pericardial membrane, while care was taken to not pierce the membrane. Two sutures (Clinisut® silk suture; 3–0) were attached to the logger which was inserted with the transparent-epoxied part, where the temperature sensor is located, pointing posteriorly. The afferent suture was placed through the designated suture hole, while the other was secured to the posterior end via a suture wrap.

For the anaesthetized baseline treatment, fish were placed in an upright foam sling in 250-L glass tanks connected to an 800-L recirculation sump. The tanks had two inflows, one to regulate water temperature and the other inserted into the mouth to create flow over the gills to maintain fish respiration. Water temperature was maintained at the same temperature as that from the holding system and dosed with 0.2 mL L^−1^ of 2-phenoxyethanol to ensure anaesthesia for the duration of the 1-h-long treatment. For the free-swimming treatment, following surgery fish were released back into the 5900-L holding tanks with conspecifics. Recovery time was monitored, and fish were released once they had regained equilibrium and showed signs of vigour. They were recaptured 1 h after recovery and sacrificed with a lethal dose (0.5 mL L^−1^) of 2-phenoxyethanol prior to recovery of the logger whereupon orientation of the device was confirmed for all individuals.

### Data processing and analysis

Data processing involved removing measurements recorded prior to recovery (once core temperature matched water temperature and erratic heart rates had subsided) and before collection (indicated by erratic heart rates associated with capture or handling). Data analysis was performed by calculating the proportion of good (QI ≤ 1) *f*_H_ readings per trial by dividing the count of 0 and 1 QI values by the total number of values. To determine if logger orientation influenced *f*_H_ accuracy, we implemented a beta regression with the proportion of good QI values as the dependent variable and logger orientation as the independent variable within the *betareg* package (Cribari-Neto and Zeileis 2010) for free-swimming fish only. We used Tukey’s post hoc tests to determine which logger orientation positions were significantly different. The small sample size for anaesthetized fish prevented any statistical testing. Median *f*_H_ are presented throughout due to the distribution of the data with a number of extreme outliers skewing means. All analyses were conducted in R version 3.5.1 (R Core Team 2018).

Captured ECG data traces were used to manually validate heart rate calculations using the software Pattern Finder (v. 1.14.0, Star-Oddi, Iceland). Intervals between the QRS waveform were measured using a double cursor that outputs the interval in beats per minute. Manually derived *f*_H_ were then compared with those calculated by the biologger.

## Results

A total of 28 fish were operated on ranging from 260 to 368 mm FL, corresponding to 500–1400 g, with no significant difference in length among treatments. Post-surgery recovery ranged from 1 to 9 min with an average of 4.5 min before equilibrium was regained and either active swimming took place or opercular beats became rhythmic. Time to recovery was positively related to size (Spearman’s rank correlation: *r*_S_ = 0.62, *P* < 0.01, *N* = 18), with larger individuals typically taking longer to recover from the effects of anaesthesia.

ECG traces showed consistent differences among orientations, with side and up typically being very similar while those from the down position were not sensible. R-wave amplitude, the positive inflection of the QRS complex associated with ventricular contraction, was greatest for the up orientation although this position also exhibited greater noise (Fig. 2b). ECGs from the side orientation exhibited lower R-wave amplitude although reduced signal noise resulted in a greater proportion of good results (low QI). ECGs from the down position consistently displayed elevated T waves, signal associated with ventricular relaxation, which likely resulted in the elevated *f*_H_ values as T-waves were falsely identified as R-waves. Manually derived *f*_H_, by measuring R–R interval distance, consistently agreed with beat per minute values automatically calculated by the logger.

**Figure 3:**
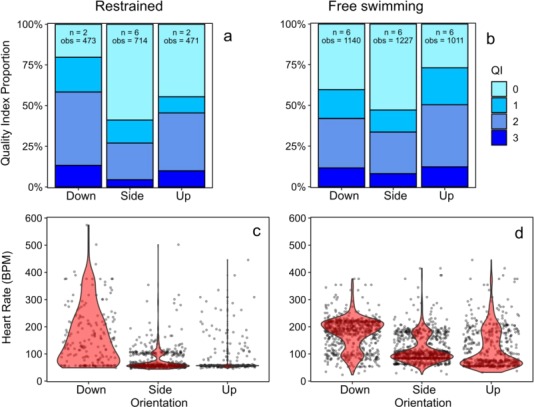
Proportion of Quality Index (QI) values and associated heart rate (BPM) recordings (QI values of 0 and 1 only) per orientation for baseline/restrained (**a** and **c**, respectively), and captive free-swimming (**b** and **d**, respectively) *Pachymetopon grande.*

Quality index scores differed greatly among logger orientations, as did the distribution of heart rate values (BPM) with side orientation consistently producing a higher proportion of good results when compared to other orientations (Fig. 3). Results for baseline treatments showed a higher proportion of 0 and 1 QI values associated with the side orientation while proportions were lower for up and dominated by poor values for the down position (Fig. 3a). Likewise, associated heart rate values varied among orientations with both side and up producing relatively low variance around a similar median of 59 and 57 BPM, respectively (Fig. 3c). The downward orientation produced unusable results with a high proportion of extreme values.

**Figure 4:**
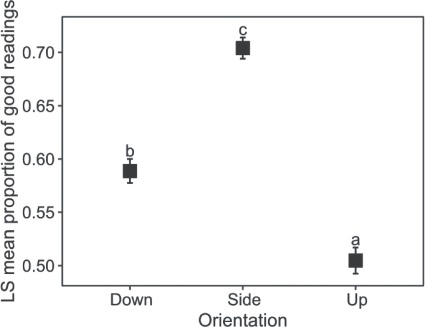
Beta-logistic regression per orientation for free-swimming *Pachymetapon grande* indicating proportion of good readings (Quality Index ≤ 1) with confidence intervals, letters denote results from a Tukey’s post hoc test.

For the free-swimming treatment, there was a significant difference (*X*^2^ (2, *N* = 18) = 809.63, *P* < 0.001) among the proportion of good values recorded per logger orientation (Fig. 4). Side orientation again produced the highest proportion of good results (QI of 0 and 1) at 66% of total recordings while down and up orientations progressively tended toward poorer QIs of 58 and 50%, respectively (Fig. 3b). Side and up orientations also captured better heart rate values, although medians differed moderately with 99 and 79 BPM, respectively. This moderate variation between orientations is most likely attributed to individual *f*_H_ variation, which differed greatly among individuals, rather than to ECG detection of individual loggers among orientations. Median values for side ranged from 67 to 182 BPM, with up similarly ranging from 56 to 165 BPM. Assessment of individual *f*_H_ typically displayed little variation around median values, although means were skewed due to few unrealistic elevated values. Median *f*_H_ values recorded from the down position were unrealistic, ranging from 120 to 215 BPM and further showing great variation around a median value.

## Discussion

Studies using and detailing biologger attachment or positioning have typically focussed on salmonids (Clark *et al*. 2009; Norling 2017; Brijs *et al*. 2018) and gadoids (Neat *et al*. 2006; Righton *et al*. 2006; Bjarnason *et al*. 2019; Davidsen *et al*. 2019) where, due to the specific anatomy of these fishes, incisions have been made along the ventral midline to access the peritoneal cavity. The skeletal system, specifically the pelvic girdle, of many perciform species prevents ventral access to the heart for biologger placement, therefore requiring a different approach. We found that for a typical sparid, *Pachymetopon grande*, the ventrolateral surgical implantation of the biologger immediately posterior to the scapulocoracoid of the pelvic girdle ensured a high consistency of successful implantations and enabled placing the logger near the pericardium, with little chance of perforation of the pericardial membrane. Furthermore, there appeared to be no damage to musculature as fish maintained the use of pectoral and ventral fins and showed no swimming irregularities after surgery.

Proper placement of R-wave detectors is critical in order to maximize the clarity and size of the ECG signal (Ponganis 2007; Cooke *et al*. 2016). Double recordings may be attributed to the detection of elevated peaks associated with atrial contraction or ventricular relaxation, P or T waves, respectively, which are both positive inflections of the ECG signal, and are noted as a possible source of error in R-wave detectors (Ponganis 2007). Analysis of the ECG signal from the down position indeed verified elevated T waves in the current study, indicating poor electrode positioning. Cooke *et al*. (2002) recommended that the placement of the electrodes should be near the pericardial cavity but perforation may result in altered cardiac performance. Based on recorded ECG signals, both side and up orientations had satisfactory electrode placement indicated by defined peaks associated with the QRS complex. Side orientation had the greatest number of successful readings, with 66% of data classified as good (QI of 0 and 1), compared to up and down, with 50 and 58% respectively. Movement of the device within the body cavity may also produce poor recordings. This is of particular concern when considering the deep body cavity of perciform fishes and therefore the double suture technique employed was considered necessary to ensure that the electrodes maintain position throughout deployment which was confirmed upon their retrieval. This appears unnecessary with fusiform-shaped fishes where measurements are typically more successful with ratios of good readings of 84% (Bjarnason *et al*. 2019) and 89% (Davidsen *et al*. 2019) for gadoids using a similar device (Star-Oddi, DST Milli-HRT). The median *f*_H_ of anaesthetized fish for both side and up orientations was very similar at 59 and 57 BPM, respectively. These values are higher than those of resting sockeye salmon *Oncorhynchus nerka* averaging 43 BPM at 19°C (Prystay *et al*. 2017) or routine rates of ~40 BPM at 18 C for smallmouth bass *Micropterus dolomieu* (Prystay *et al*. 2019). Our values did, however, more closely approximate those recorded for resting centrarchid species, pumpkinseed *Lepomis gibbosus* and bluegill *L. macrochirus* averaging ~60 BPM at 18 C (Cooke *et al*. 2002, 2010). Free-swimming rates were notably higher than those of anaesthetized fish. Although there was considerable difference between median values of side and up orientations this was most likely due to individual variation rather than differences between orientation and R–R signal strength, as was confirmed by ECG traces. Bjarnason *et al*. (2019) similarly found notable individual variation in *f*_H_ for *Gadus morhua* during a 5-week-long experimental period, with means ranging from 29.9 to 48.3 BPM at 10 C. Free-swimming rates were also comparable to those attained for centrarchids, *L. gibbosus* and *L. macrochirus* after exhaustive exercise (~100 BPM at 18 C (Cooke *et al*. 2010)) and active swimming yellowtail kingfish *Seriola lalandi* (95 BPM at 20 C (Clark *et al*. 2006)). While peak *f*_H_ of stressed *O. nerka* averaged 105 BPM at 19 C following simulated fisheries catch and release (Prystay *et al*. 2017). It should be noted, however, that our recorded *f*_H_ are likely elevated as stressed animals may not have the same capacity to control their *f*_H_ as an unstressed animal (Brijs *et al*. 2018) and insufficient recovery times following anaesthesia and surgery has been suggested to mask *f*_H_ modulation of fishes (Clark *et al*. 2005). Heart rate of *G. morhua*, for example, remained elevated for 8–10 days following surgery (Webber *et al*. 1998), while rainbow trout *O. mykiss* required ~4 days to regain normal activity (Brijs *et al*. 2018).

While the measurement of cardiac output and its relationship with metabolism is not a new field of study, the availability of a commercially available device which is fully implantable does present a new set of opportunities. Longer-term studies are indicating that cardiac output is less affected by changes in stroke volume than previously suspected, which may be due to insufficient recovery periods, indicating that *f*_H_ is a reliable indicator of energy expenditure (Clark *et al*. 2005; Cooke *et al*. 2016). Contemporary studies using modern *f*_H_ loggers are shedding light on the physiology of fish in response to temperature (Ekström *et al*. 2018, 2019; Skeeles *et al*. 2020), fisheries catch and release (Prystay *et al*. 2017), aquaculture stress (Brijs *et al*. 2018, 2019) and parental care investment (Prystay *et al*. 2019). They have the potential to contribute notably to bioenergetics modelling (Cooke *et al*. 2016) as well as provide insights into physiological responses to environmental change and stressors while also illuminating the mechanisms involved in adapting to these changes (Norin and Metcalfe 2019).

Our intention was to describe an optimal implantation method and evaluate the effect of orientation or electrode placement on data quality of a commercially available *f*_H_ logger for a typical perciform fish. We found that the ventrolateral incision enabled placement of the device near the heart, was minimally invasive, offered quick surgery time and did not require extensive surgical training. Orientation of the electrodes was critical for data quality and the three orientations tested produced exceptionally poor to good results. In contrast with our findings, orientation of electrodes was found to have no impact on data quality for rainbow trout as long as the logger was placed as close to the heart as possible (Norling 2017). We advise that for studies on perciform fish using similar devices as that of the current study, loggers should be placed or moved as close to the heart as possible, electrodes should be in contact with musculature and double sutures should be used to prevent risk of movement or displacement. Prior to any investigation using biologgers, researchers should conduct pilot experiments to determine optimal placement of the devices. Trial experiments using both resting/anaesthetized and active fish will also assist with data analysis by determining *f*_H_ threshold limits, values falling outside of these limits can therefore be excluded from further analysis.

## Funding

This work was supported by a National Research Foundation (NRF) South Africa research development grant for Y-rated researchers (RDYR14071676426). This research forms part of a larger project funded by the Rhodes University Sandisa Imbewu Fund and falls within the Aquatic Eco-Physiology Research Platform (AERP) of the South African Institute for Aquatic Biodiversity (SAIAB).

## References

[ref1] Anttila K, Jørgensen SM, Casselman MT, Timmerhaus G, Farrell AP, Takle H (2014) Association between swimming performance, cardiorespiratory morphometry, and thermal tolerance in Atlantic salmon (*Salmo salar* L.). Front Mar Sci 1: 76.

[ref2] Bjarnason A, Gunnarsson A, Árnason T, Oddgeirsson M, Sigmarsson AB, Gunnarsson Á (2019) Alidation of ECG-derived heart rate recordings in Atlantic cod (*Gadus morhua* L.) with an implantable data logging system. Anim Biotelemetry 7: 13.

[ref3] Brijs J, Sandblom E, Axelsson M, Sundell K, Sundh H, Huyben D, Broström R, Kiessling A, Berg C, Gräns A (2018) The final countdown: continuous physiological welfare evaluation of farmed fish during common aquaculture practices before and during harvest. Aquaculture 495: 903–911.

[ref4] Brijs J, Sandblom E, Axelsson M, Sundell K, Sundh H, Kiessling A, Berg C, Gräns A (2019) Remote physiological monitoring provides unique insights on the cardiovascular performance and stress responses of freely swimming rainbow trout in aquaculture. Sci Rep 9: 9090. 31235773 10.1038/s41598-019-45657-3 PMC6591390

[ref5] Butler PJ, Green JA, Boyd IL, Speakman JR (2004) Measuring metabolic rate in the field: the pros and cons of the doubly labelled water and heart rate methods. Funct Ecol 18: 168–183.

[ref6] Clark TD, Hinch SG, Taylor BD, Frappell PB, Farrell AP (2009) Sex differences in circulatory oxygen transport parameters of sockeye salmon (*Oncorhynchus nerka*) on the spawning ground. J Comp Physiol B 179: 663–671. 19252914 10.1007/s00360-009-0349-1

[ref7] Clark TD, Ryan T, Ingram BA, Woakes AJ, Butler PJ, Frappell PB (2005) Factorial aerobic scope is independent of temperature and primarily modulated by heart rate in exercising Murray cod (*Maccullochella peelii peelii*). Physiol Biochem Zool 78: 347–355. 15887081 10.1086/430034

[ref8] Clark TD, Sandblom E, Hinch SG, Patterson DA, Frappell PB, Farrell AP (2010) Simultaneous biologging of heart rate and acceleration, and their relationships with energy expenditure in free-swimming sockeye salmon (*Oncorhynchus nerka*). J Comp Physiol B 180: 673–684. 20063165 10.1007/s00360-009-0442-5

[ref9] Clark TD, Seymour RS, Forster ME, Farrell AP (2006) Cardiorespiratory physiology and swimming energetics of a high-energy-demand teleost, the yellowtail kingfish (*Seriola lalandi*). J Exp Biol 209: 3940–3951. 16985209 10.1242/jeb.02440

[ref10] Cooke SJ, Brownscombe JW, Raby GD, Broell F, Hinch SG, Clark TD, Semmens JM (2016) Remote bioenergetics measurements in wild fish: opportunities and challenges. Comp Biochem Physiol Part A Mol Integr Physiol 202: 23–37. 10.1016/j.cbpa.2016.03.022 27063208

[ref11] Cooke SJ, Bunt CM, Schreer JF, Philipp DP (2002) Attachment, validation, and preliminary deployment of ultrasonic heart rate transmitters on largemouth bass, *Micropterus salmoides*. Aquat Living Resour 15: 155–162.

[ref12] Cooke SJ et al. (2012) Conservation physiology in practice: how physiological knowledge has improved our ability to sustainably manage Pacific salmon during up-river migration. Philos Trans R Soc B Biol Sci 367: 1757–1769. 10.1098/rstb.2012.0022 PMC3350662 22566681

[ref13] Cooke SJ, Hinch SG, Wikelski M, Andrews RD, Kuchel LJ, Wolcott TG, Butler PJ (2004) Biotelemetry: a mechanistic approach to ecology. Trends Ecol Evol 19: 334–343. 16701280 10.1016/j.tree.2004.04.003

[ref14] Cooke SJ, Schreer JF, Wahl DH, Philipp DP (2010) Cardiovascular performance of six species of field-acclimatized centrarchid sunfish during the parental care period. J Exp Biol 213: 2332–2342. 20543132 10.1242/jeb.030601

[ref15] Cribari-Neto F, Zeileis A (2010) Beta regression in *R*. J Stat Softw 34: 1–24.

[ref16] Davidsen JG et al. (2019) Effects of sound exposure from a seismic airgun on heart rate, acceleration and depth use in free-swimming Atlantic cod and saithe. Conserv Physiol 7 1 1–19 doi: 10.1093/conphys/coz020. PMC6521782 31110769

[ref17] Donaldson MR, Clark TD, Hinch SG, Cooke SJ, Patterson DA, Gale MK, Frappell PB, Farrell AP (2010) Physiological responses of free-swimming adult coho salmon to simulated predator and fisheries encounters. Physiol Biochem Zool 83: 973–983. 20961224 10.1086/656336

[ref18] Ekström A, Axelsson M, Gräns A, Brijs J, Sandblom E (2018) Importance of the coronary circulation for cardiac and metabolic performance in rainbow trout (*Oncorhynchus mykiss*). Biol Lett 14 17 1–4 doi: 10.1098/rsbl.2018.0063. PMC6083225 30045901

[ref19] Ekström A, Gräns A, Sandblom E (2019) Can’t beat the heat? Importance of cardiac control and coronary perfusion for heat tolerance in rainbow trout. J Comp Physiol B Biochem Syst Environ Physiol 189: 757–769. 10.1007/s00360-019-01243-7 31707423

[ref20] Farrell AP, Eliason EJ, Sandblom E, Clark TD (2009) Fish cardiorespiratory physiology in an era of climate change. Can J Zool 87: 835–851.

[ref21] Graham AL, Cooke SJ (2008) The effects of noise disturbance from various recreational boating activities common to inland waters on the cardiac physiology of a freshwater fish, the largemouth bass (*Micropterus salmoides*). Aquat Conserv Mar Freshw Ecosyst 18: 1315–1324.

[ref23] Heemstra PC, Heemstra E (2004) Coastal Fishes of Southern Africa. NISC (PTY) LTD Grahamstown, South Africa.

[ref24] Lucas MC, Johnstone ADF, Priede IG (1993) Use of physiological telemetry as a method of estimating metabolism of fish in the natural environment. Trans Am Fish Soc 122: 822–833.

[ref25] Lucas MC, Priede IG, Armstrong JD, Gindy ANZ, Vera L (1991) Direct measurements of metabolism, activity and feeding behaviour of pike, *Esox Zucius* L., in the wild, by the use of heart rate telemetry. J Fish Biol 39: 325–345.

[ref26] Neat FC, Wright PJ, Zuur AF, Gibb IM, Gibb FM, Tulett D, Righton DA, Turner RJ (2006) Residency and depth movements of a coastal group of Atlantic cod (*Gadus morhua* L.). Mar Biol 148: 643–654.

[ref27] Norin T, Metcalfe NB (2019) Ecological and evolutionary consequences of metabolic rate plasticity in response to environmental change. Philos Trans R Soc B Biol Sci 374: 20180180. 10.1098/rstb.2018.0180 PMC6365862 30966964

[ref28] Norling TA (2017) Remotely Monitoring Heart-Rate and Feeding Behaviour of Fish by Using Electronic Sensor-Tags. Swedish University of Agricultural Sciences, Umea.

[ref29] Ponganis PJ (2007) Bio-logging of physiological parameters in higher marine vertebrates. Deep Sea Res Part II Top Stud Oceanogr 54: 183–192.

[ref30] Priborsky J, Velisek J (2018) A review of three commonly used fish anesthetics. Rev Fish Sci Aquac 26: 417–442.

[ref31] Prystay TS (2018) Exploring the Relationship between Physiological Performance and Reproductive Investment in Wild Fish Using Heart Rate Biologgers. Carleton University, Ottawa, Canada.

[ref32] Prystay TS, Eliason EJ, Lawrence MJ, Dick M, Brownscombe JW, Patterson DA, Crossin GT, Hinch SG, Cooke SJ (2017) The influence of water temperature on sockeye salmon heart rate recovery following simulated fisheries interactions. Conserv Physiol 5, 5: 1–12. doi: 10.1093/conphys/cox050. PMC5597901 28928974

[ref33] Prystay TS, Lawrence MJ, Zolderdo AJ, Brownscombe JW, Bruijn R de, Eliason EJ, Cooke SJ (2019) Exploring relationships between cardiovascular activity and parental care behavior in nesting smallmouth bass: a field study using heart rate biologgers. Comp Biochem Physiol-Part A Mol Integr Physiol 234: 18–27. 10.1016/j.cbpa.2019.04.012 31004808

[ref34] R Core Team (2018) R: A Language and Environment for Statistical Computing.

[ref35] Righton D, Kjesbu OS, Metcalfe J (2006) A field and experimental evaluation of the effect of data storage tags on the growth of cod. J Fish Biol 68: 385–400.

[ref36] Schreer JF, Cooke SJ, McKinley RS (2001) Cardiac response to variable forced exercise at different temperatures: an angling simulation for smallmouth bass. Trans Am Fish Soc 130: 783–795.

[ref37] Skeeles MR, Winkler AC, Duncan MI, James NC, Walt K-A van der, Potts WM (2020) The use of internal heart rate loggers in determining cardiac breakpoints of fish. J Therm Biol 102524: 1–17. 10.1016/j.jtherbio.2020.102524 32364965

[ref38] Thorarensen H, Gallaugher PE, Farrell AP (1996) The limitations of heart rate as a predictor of metabolic rate in fish. J Fish Biol 49: 226–236.

[ref39] Webber DM, Boutilier RG, Kerr SR (1998) Cardiac output as a predictor of metabolic rate in cod *gadus morhua*. J Exp Biol 201: 2779–2789. 9732332 10.1242/jeb.201.19.2779

